# Effectiveness of a Nurse-Led Intervention on Pre-procedural Anxiety Among Patients Undergoing Endoscopy: A Quasi-experimental Trial

**DOI:** 10.7759/cureus.93166

**Published:** 2025-09-25

**Authors:** Vikas Chaudhary, Suresh Sharma, Ashok Kumar, Ashish Agarwal, Chhagan L Birda, Khina Sharma, Himanshu Vyas, Anoop Sharma, Ramesh Kumar

**Affiliations:** 1 College of Nursing, All India Institute of Medical Sciences, Jodhpur, IND; 2 Gastroenterology, All India Institute of Medical Sciences, Jodhpur, IND

**Keywords:** anxiety, endoscopy, nurse-led intervention, nursing, pre-procedural anxiety

## Abstract

Introduction: Procedural anxiety is the fear or worry associated with medical procedures that may interfere with the ability to receive appropriate care. It can lead to heightened pain perception, greater sedation requirements, reduced cooperation, and compromised procedural safety and patient satisfaction.

Methods: A quantitative, quasi-experimental post-test-only design was employed among 120 patients undergoing endoscopy, selected through a non-probability convenience sampling technique. Anxiety levels were assessed using the Hamilton Anxiety Rating Scale.

Results: The study findings revealed a mean difference of 8.31 (95% CI: 6.10-10.52) between the control and experimental groups, indicating that the nurse-led intervention was effective in reducing pre-procedural anxiety among patients undergoing endoscopy. In the experimental group, pre-procedural anxiety showed a significant association with the patient’s educational level, dietary pattern, purpose of the endoscopic procedure, and previous exposure. In contrast, in the control group, significant associations were observed with dietary pattern, patient’s diagnosis, previous hospitalization, purpose of the endoscopic procedure, and previous exposure.

Conclusion: Nurse-led interventions significantly reduced pre-procedural anxiety among endoscopy patients. Integrating such approaches to patient groups particularly prone to anxiety offers a promising pathway to enhance overall patient experience and procedural success.

## Introduction

Endoscopic procedures, encompassing both diagnostic and therapeutic applications, are routinely employed in the assessment and management of gastrointestinal (GI) disorders. Among these, upper and lower gastrointestinal endoscopies are the most frequently performed interventions in clinical practice [1].

Despite the clinical benefits, endoscopic procedures are often associated with heightened levels of anxiety and psychological distress among patients. This procedural anxiety, characterized by fear, apprehension, and emotional discomfort, may negatively impact patient cooperation, satisfaction, and overall procedural outcomes. Individuals scheduled for endoscopy frequently report stress related to the invasive nature of the procedure, the anticipation of pain, and unfamiliarity with the medical environment [2,3].

To address this challenge, a variety of non-pharmacological interventions have been explored to reduce anxiety prior to invasive procedures. These include mental health counseling, audiovisual educational materials, music therapy, peer support interactions, and familiarization with the healthcare team and equipment [4]. Although sedation is commonly used to ease discomfort during endoscopy, it does not fully eliminate the psychological burden experienced beforehand.

Recent literature underscores the importance of identifying effective strategies that can be implemented pre-procedurally to alleviate anxiety, particularly those that are cost-effective and feasible in routine clinical settings. Nurse-led interventions have emerged as promising approaches due to their accessibility, personalized care, and potential to establish trust and communication between patients and healthcare providers [5,6].

This quasi-experimental study was undertaken to assess the effectiveness of a nurse-led intervention in reducing pre-procedural anxiety among patients undergoing endoscopy at a tertiary care institute in western India. By evaluating patient experiences and measuring anxiety outcomes, the study aims to contribute to evidence-based practices that enhance patient comfort and satisfaction in endoscopic care.

## Materials and methods

A quasi-experimental post-test-only design, utilizing a quantitative research approach, was employed to evaluate the effectiveness of nurse-led interventions on pre-procedural anxiety among patients undergoing endoscopy. This study was conducted in the Endoscopy Unit of All India Institute of Medical Sciences (AIIMS), Jodhpur, Rajasthan, India. 

Patients aged 18 years and above who were able to understand either Hindi or English and were willing to provide informed consent were included in the study. Patients with cognitive impairment or those who were sedated or unconscious prior to assessment were excluded.

Participants were recruited through non-probability convenience sampling between December 2023 and February 2024. Eligible patients were those scheduled to undergo diagnostic or therapeutic endoscopic procedures during this period. After verifying eligibility, those who consented were enrolled in the study. To minimize potential confounding, recruitment of the control group was completed prior to initiating enrolment for the experimental group.

Patients in the control group received routine care, which consisted of a brief explanation of the procedure provided by the healthcare team. In contrast, patients in the experimental group received both routine care and a structured nurse-led pre-procedural counselling session.

The intervention was administered by a graduate nursing officer who was specifically trained in patient counselling. The session was based on a predefined counselling format. Each participant in the experimental group received a one-to-one counselling session delivered by the same nursing officer to ensure consistency. Each session lasted approximately 10-15 minutes and was conducted prior to the procedure. The counselling covered key information such as the brief explanation of the procedure, absence of pain though mild throat sensitivity may occur, and reassurance regarding the use of throat spray and light sedation for comfort. Patients were informed that the endoscope is slim, smooth, and does not obstruct breathing. They were reassured that normal respiration is maintained, vital signs are continuously monitored, oxygen is supplemented as needed, and biopsies, if required, are performed painlessly. Patients were also advised that they could resume oral intake approximately one hour post-procedure.

Baseline sociodemographic and clinical data were collected for all participants. These mainly included age, gender, educational status, dietary pattern, diagnosis, and indication for endoscopy.

Pre-procedural anxiety was measured using the Hamilton Anxiety Rating Scale (HAM-A). The HAM-A is a validated, open-access tool consisting of 14 items rated on a five-point Likert scale (0-4), with total scores ranging from 0 to 56. Anxiety severity is categorized as follows: no anxiety (0), mild (1-14), moderate (15-28), severe (29-42), and very severe (>43) [7]. In the control group, anxiety levels were assessed once before the endoscopic procedure. In the experimental group, anxiety assessments were carried out 10 minutes after the nurse-led intervention. The tool was administered by a trained nursing officer who was familiar with the use of HAM-A.

Ethical approval for the study was obtained from the Institutional Ethics Committee of AIIMS, Jodhpur (Reference Number: AIIMS/IEC/2022/4242, dated 09/11/2022). All participants were fully informed about the objectives, procedures, confidentiality measures, and their right to withdraw from the study at any time without compromising their medical care. Written informed consent was obtained prior to enrolment. Strict measures were taken to ensure confidentiality and anonymity throughout the research process.

The sample size of the study was calculated for comparison of two independent groups using the standard formula for two-sample means:

$n (per group)=2 (Z1&minus;&alpha;/2+Z1&minus;&beta;)2/d2$

where Z1−α/2 = 1.96 (α = 0.05, two-tailed), Z1−β = 0.842 (80% power), and d is the standardized effect size (Cohen’s d) [8]. Thus, assuming a standardized effect size of d ≈ 0.51, the estimated sample size is 60 participants per group (total n = 120). Based on these considerations, the study enrolled 120 participants (60 in the nurse-led intervention group and 60 in the control group). 

Data were analyzed using the IBM SPSS Statistics for Windows, Version 20.0 (Released 2011; IBM Corp., Armonk, NY, USA). Descriptive statistics (mean, standard deviation, frequencies, and percentages) were used to summarize the data. For inferential analysis, the t-test was applied to compare independent quantitative variables, and the chi-square test was used to examine associations between categorical variables. A p-value of <0.05 was considered statistically significant and <0.001 as highly significant.

## Results

**Figure 1:**
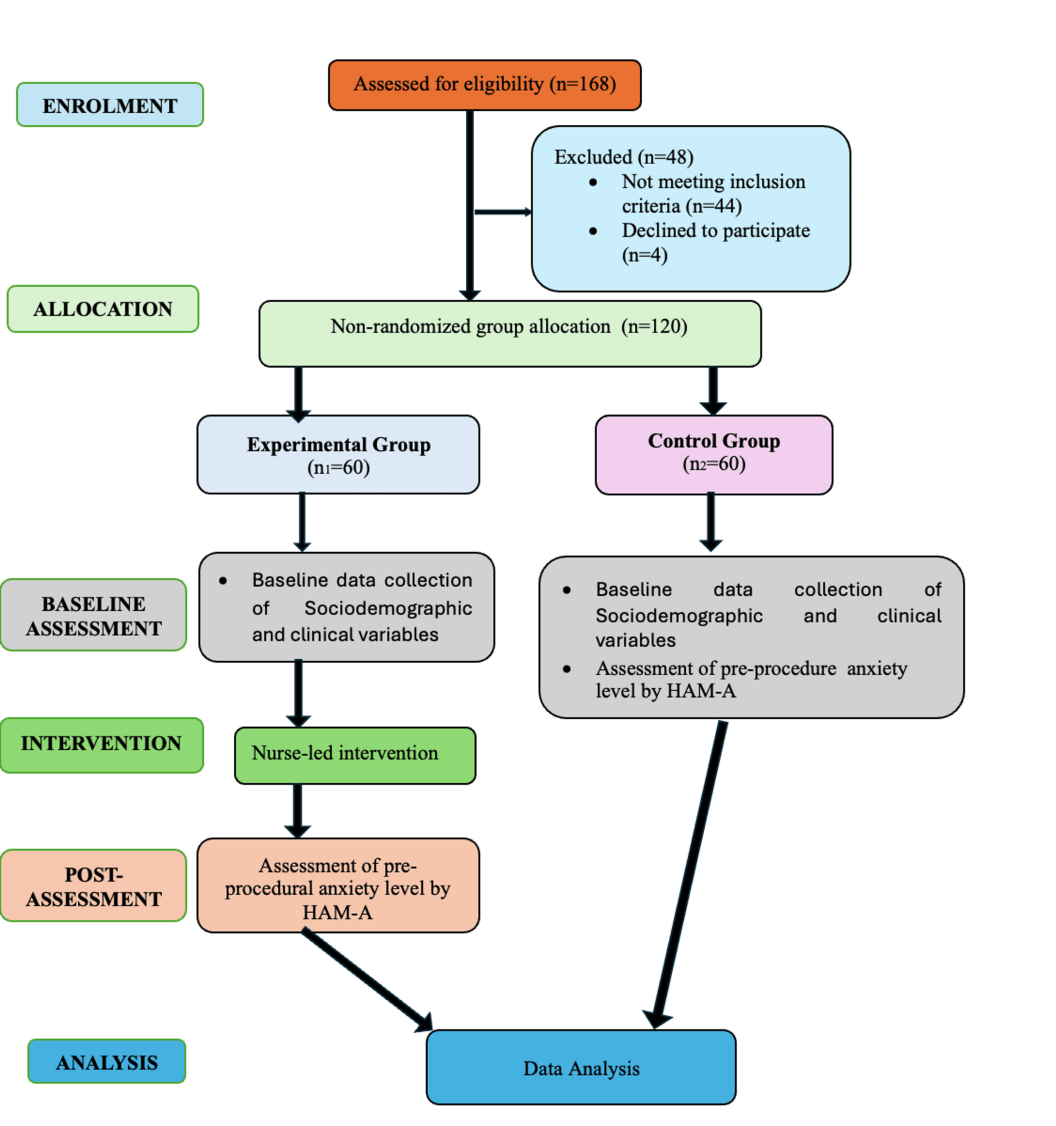
CONSORT flowchart of the study

Figure 1 presents the Consolidated Standards of Reporting Trials (CONSORT) flowchart of the study.

Table 1 presents the distribution and comparison of sociodemographic and clinical characteristics between the experimental and control groups. The two groups were statistically comparable across most variables. However, a significant difference was observed in the purpose of the endoscopic procedure (p = 0.03), with a higher proportion of participants in the experimental group undergoing the procedure for diagnostic purposes (96.7%) compared to the control group (83.3%).

**Table 1:** Comparison of sociodemographic and clinical variables between experimental and control groups (N = 120) *Chi-square test; p-value significant at <0.05.

	Sociodemographic variables	Experimental group, f (%)	Control group, f (%)	p-value
Age	18-30 years	11 (18.3)	14 (23.3)	0.82
	31-45 years	16 (26.7)	17(28.3)	
	46-60 years	16 (26.7)	16(26.7)	
	More than 60 years	17 (28.3)	13 (21.7)	
Educational level	Illiterate	23 (38.3)	22 (36.7)	0.51
	Primary school	21 (35)	15 (25)	
	High school	8 (13.3)	12 (20)	
	Graduate and above	8 (13.3)	11 (18.3)	
Gender	Male	35 (58.3)	31 (51.7)	0.58
	Female	25 (41.7)	29 (48.3)	
Dietary pattern	Pure vegetarian	42 (60)	47 (78.3)	0.11
	Egg vegetarian	4 (6.7)	0	
	Non-vegetarian	14 (23.3)	13 (21.7)	
Indication for endoscopy	Disorder of the esophagus	11 (18.3)	4 (6.7)	0.25
	Disorder of the stomach	39 (65)	42 (70)	
	Disorder of the duodenum	1 (1.7)	2 (3.3)	
	Others	9 (15)	12(20)	
Previous hospitalization	Yes	24 (40)	25 (41.7)	0.99
	No	36 (60)	35 (58.3)	
Purpose of endoscopic procedure	Diagnostic	58 (96.7)	50 (83.3)	0.03
	Therapeutic	2 (3.3)	10 (16.7)	
Previous endoscopy	Yes	22 (36.7)	21 (35)	0.99
	No	38 (63.3)	39 (65)	

**Figure 2:**
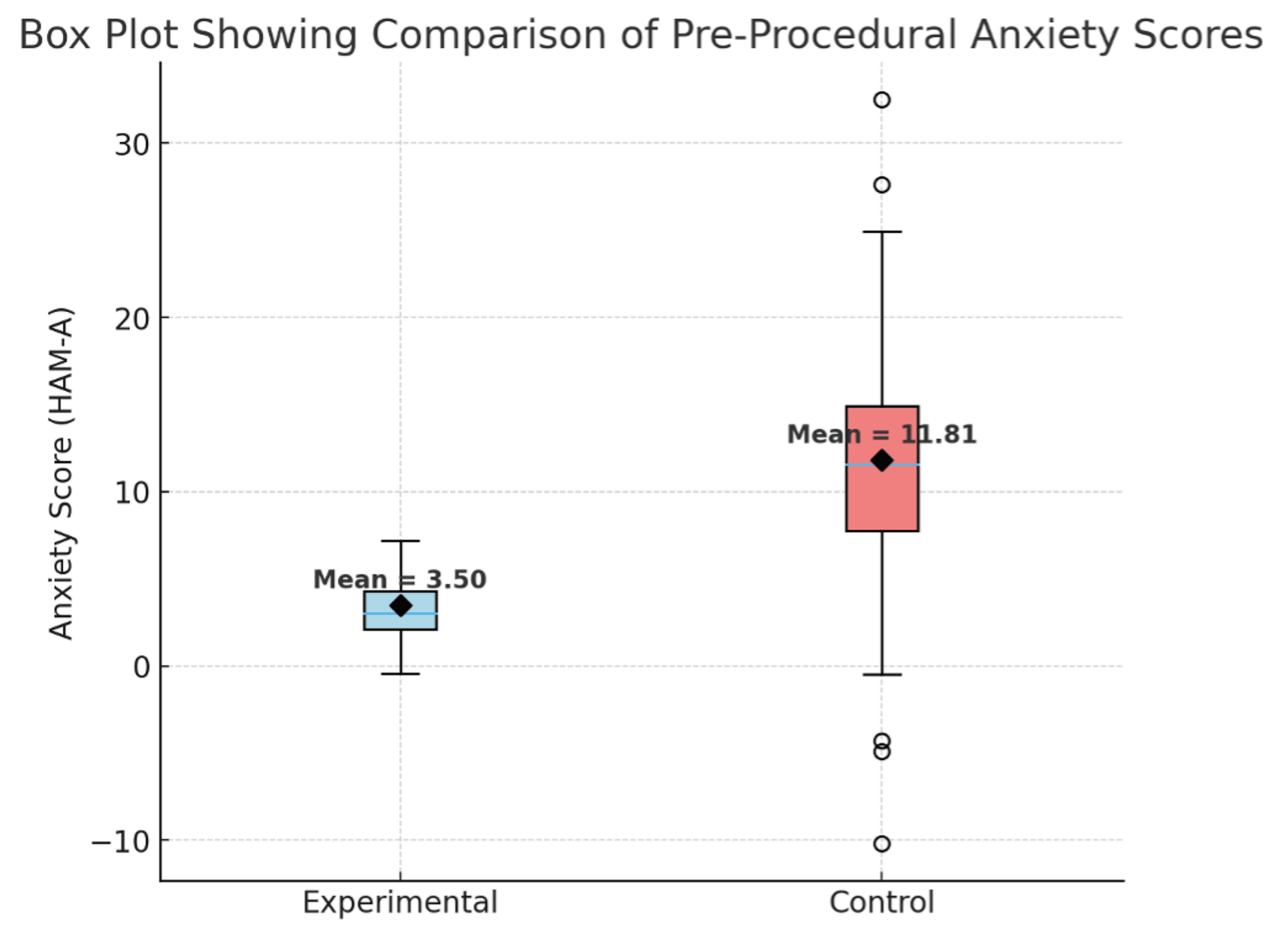
Box plot showing mean values of pre-procedural anxiety in both groups

Figure 2 shows that the mean pre-procedural anxiety score in the experimental group (mean ± SD = 3.50 ± 1.99) was substantially lower than in the control group (mean ± SD = 11.81 ± 8.40). A statistically significant difference between the two groups (p < 0.001) was observed. The mean difference of 8.31 (95% CI: 6.10-10.52) indicates that the nurse-led intervention was effective in reducing pre-procedural anxiety among patients undergoing endoscopy. 

After the nurse-led intervention, in the experimental group, all participants (100%) had mild anxiety, with no cases of moderate, severe, or very severe anxiety. In the control group, 76.67% had mild anxiety, 20% had moderate anxiety, and 3.33% had severe anxiety (Table 2). No participants in either group had no anxiety or very severe anxiety. This indicates lower anxiety levels in the experimental group compared to the control group. 

**Table 2:** Level of pre-procedural anxiety among control and experimental groups Maximum score: 56. Minimum score: 0.

Level of pre-procedural anxiety	Frequency, f (%)	
	Experimental group	Control group
No anxiety (0)	0	0
Mild (1-14)	60 (100)	46 (76.67)
Moderate (15-28)	0	12 (20)
Severe (29-42)	0	2 (3.33)
Very severe (>43)	0	0

The association between pre-procedural anxiety and selected sociodemographic variables was examined for both groups. In both the experimental and control groups, dietary pattern and the purpose of the endoscopic procedure showed a significant association with anxiety. Within the experimental group, additional significant associations were identified with educational status and history of previous endoscopy. In the control group, significant relationships were also found with the indication for endoscopy and prior hospitalization. Conversely, no significant association was observed with age or gender in either group.

## Discussion

The present study was undertaken to evaluate the effectiveness of a nurse-led intervention in reducing pre-procedural anxiety among patients undergoing endoscopy. The findings demonstrated a statistically significant difference in the mean levels of anxiety between the experimental and control groups, indicating that the nurse-led intervention was effective in alleviating anxiety prior to the procedure. This supports the premise that structured educational approaches led by nurses can play a crucial role in reducing procedural anxiety.

These findings align with those of Behrouzian et al. [9], who investigated the impact of psychological preparation on anxiety among patients undergoing upper gastrointestinal endoscopy. In their study, participants in the experimental group received both informational and behavioral interventions. Initially, no significant differences were observed between the groups regarding state and trait anxiety (p > 0.05); however, following the intervention, there was a notable reduction in both anxiety measures in the experimental group (p < 0.05). The authors concluded that psychological preparation significantly reduced anxiety levels, reinforcing the efficacy of such interventions in clinical settings [9].

Further support is provided by another study [10] that examined the effect of video-assisted teaching on anxiety among patients scheduled for upper GI endoscopy. The study reported a statistically significant reduction in anxiety levels after the intervention in both experimental and control groups (p < 0.001). Interestingly, the difference in anxiety reduction between the groups was not statistically significant post-intervention, suggesting that both verbal information and video-assisted teaching can be equally effective in mitigating procedural anxiety [10].

In addition to assessing the effectiveness of the intervention, the present study explored associations between pre-procedural anxiety and various demographic and clinical variables. In the experimental group, significant associations were found with educational level, dietary habits, the purpose of the endoscopic procedure, and previous exposure to endoscopy. In the control group, anxiety levels were significantly associated with dietary habits, diagnosis, prior hospitalization, purpose of the procedure, and previous exposure. This indicates that individualized factors may influence patients' anxiety levels and should be considered when designing pre-procedural educational strategies.

Contrastingly, a significant association was found between gender and changes in anxiety levels (p > 0.01), while no significant relationships were observed with variables such as age, educational status, previous hospitalizations, or prior endoscopy experiences among family members [10]. These differing findings highlight the context-specific nature of anxiety determinants and suggest the need for tailored interventions based on patient demographics and clinical history. Also, factors such as education, previous endoscopy experience, and diagnosis may have impacted patients’ anxiety levels by shaping their knowledge, expectations, and perception of disease severity. To address this in future studies, researchers should consider strategies like stratified sampling, balanced recruitment, and statistical adjustments (e.g., regression or ANCOVA). Careful control of these variables will help ensure that differences in anxiety are attributable to the intervention rather than external influences.

Thus, the present study reinforces the importance of educational interventions, particularly those led by nurses, in effectively managing pre-procedural anxiety. Approaches such as verbal education, informational booklets, and video-assisted teaching have shown promising results. Providing appropriate, patient-centered education not only empowers individuals with knowledge but also helps reduce fear and anxiety associated with invasive procedures like endoscopy. This emphasizes the role of nurses as educators and advocates in promoting patient well-being across all backgrounds, irrespective of age, education, or cultural differences.

Limitations of the study

The findings of this study have limited generalizability as it was conducted in a single setting and did not employ a pre-test. In addition, the use of a convenience sampling technique for participant selection further restricts the external validity of the results. The researcher also had limited control over external factors influencing the experimental and control groups. Therefore, future research is recommended using true experimental designs with larger sample sizes across multiple settings to enhance generalizability. Studies incorporating pre-test and post-test designs, along with interventions such as information booklets or videos to reduce pre-procedural anxiety, may provide more robust evidence.

## Conclusions

The present study indicates that nurse-led interventions have the potential to reduce pre-procedural anxiety in patients undergoing endoscopy. Patients who received structured education and reassurance appeared more prepared and less apprehensive about the procedure. Although encouraging, these findings should be interpreted with caution due to limitations in design and sample size. Further studies with larger, more diverse groups and stronger methodologies are recommended to validate these outcomes. Even so, integrating patient education as a routine component of pre-procedural care, particularly for patients prone to anxiety, may serve as a simple and effective strategy to enhance patient comfort and cooperation.
